# Profiling Selective Packaging of Host RNA and Viral RNA Modification in SARS-CoV-2 Viral Preparations

**DOI:** 10.3389/fcell.2022.768356

**Published:** 2022-02-03

**Authors:** Noah Peña, Wen Zhang, Christopher Watkins, Mateusz Halucha, Hala Alshammary, Matthew M. Hernandez, Wen-Chun Liu, Randy A. Albrecht, Adolfo Garcia-Sastre, Viviana Simon, Christopher Katanski, Tao Pan

**Affiliations:** ^1^ Department of Molecular Genetics and Cell Biology, University of Chicago, Chicago, IL, United States; ^2^ Department of Biochemistry and Molecular Biology, University of Chicago, Chicago, IL, United States; ^3^ Department of Microbiology, Icahn School of Medicine at Mount Sinai, New York, NY, United States; ^4^ Department of Pathology, Molecular and Cell Based Medicine, Icahn School of Medicine at Mount Sinai, New York, NY, United States; ^5^ The Global Health and Emerging Pathogen Institute, Icahn School of Medicine at Mount Sinai, New York, NY, United States; ^6^ Division of Infectious Diseases, Department of Medicine, Icahn School of Medicine at Mount Sinai, New York, NY, United States; ^7^ Committee on Microbiology, University of Chicago, Chicago, IL, United States

**Keywords:** SARS-CoV-2, tRNA, modification, SRP RNA, packaging

## Abstract

Viruses package host RNAs in their virions which are associated with a range of functions in the viral life cycle. Previous transcriptomic profiling of host RNA packaging mostly focused on retroviruses. Which host RNAs are packaged in other viruses at the transcriptome level has not been thoroughly examined. Here we perform proof-of-concept studies using both small RNA and large RNA sequencing of six different SARS-CoV-2 viral isolates grown on VeroE6 cells to profile host RNAs present in cell free viral preparations and to explore SARS-CoV-2 genomic RNA modifications. We find selective enrichment of specific host transfer RNAs (tRNAs), tRNA fragments and signal recognition particle (SRP) RNA in SARS-CoV-2 viral preparations. Different viral preparations contain the same set of host RNAs, suggesting a common mechanism of packaging. We estimate that a single SARS-CoV-2 particle likely contains up to one SRP RNA and four tRNA molecules. We identify tRNA modification differences between the tRNAs present in viral preparations and those in the uninfected VeroE6 host cells. Furthermore, we find uncharacterized candidate modifications in the SARS-CoV-2 genomic RNA. Our results reveal an under-studied aspect of viral-host interactions that may be explored for viral therapeutics.

## Introduction

Viral assembly is a critical stage in the viral life cycle that produces mature virus containing the viral genome and proteins needed to infect another host target cell. As early as 1980s it was shown that certain viruses also package host RNAs into their virions. Host transfer RNA (tRNA) is a major cellular RNA family, which is packaged in virions (Isaac and Keene, 1981; Jiang et al., 1993). tRNAs are the most abundant RNA in copy numbers in cells, and their small size and stable structure make them good targets for interacting with viral RNA and viral proteins.

The best studied viral packaging of host RNAs has been described for retroviruses. Retroviruses require a specific host tRNA as reverse transcriptase primers in the cDNA synthesis of the viral genomic RNA upon infection. HIV-1 uses tRNA^Lys^(TTT) from the host cell since it has a fully complementary sequence of ∼20 nucleotides to the primer binding site of the retroviral genome (Litvak et al., 1994). In addition to tRNA^Lys^(TTT), other prominent tRNAs packaged into the virions include tRNA^Lys^(CTT), tRNA^Asn^(GTT) and others (Pavon-Eternod et al., 2010). Furthermore, retroviruses also package non-coding RNAs, the prominent one is the signal recognition particle (SRP) RNA (Eckwahl et al., 2015; Eckwahl et al., 2016). SRP RNA is a component of the SRP particle that is required for the co-translational synthesis of membrane proteins and secretory proteins (Keenan et al., 2001). However, aside from the tRNA serving as the primer for retroviral replication, the precise functions of the other packaged RNAs remain to be elucidated. One possibility is that the packaged RNAs are proximal to the cellular locations of viral assembly and encapsulation. If assembly is co-translational, the composition of packaged RNAs may reflect the translation machinery where the viral structural protein synthesis occurs. For example, the packaged tRNAs may be enriched for those reading the retroviral gag protein codons (van Weringh et al., 2011). Another possibility is that packaged host tRNAs may reduce innate immune recognition of viral genomic sequences by cytosolic pattern recognition receptors, as the host tRNAs may be seen as self-RNA upon infection (Karikó et al., 2005). However, viral packaging of non-retroviruses has rarely been explored using transcriptomic approaches which could potentially generate functional hypotheses on host RNA packaging in viral biology.

Here, we utilize both small RNA (<200 nt) and large RNA (>200 nt) sequencing to identify host RNAs that are present in cell free viral preparations which consist mostly of the SARS-CoV-2 viruses cultured on VeroE6 cells. We obtain sequencing data for six distinct primary SARS-CoV-2 isolates (Table 1) and compare them to those from uninfected cells. We identify selective enrichment of host tRNAs and SRP RNA in the viral preparations. tRNA^Lys^(TTT) is among the selectively packaged tRNAs, just like HIV-1. We find that a specific tRNA modification may influence tRNA packaging, and some packaged tRNAs are likely tRNA fragments. We also identify a low level of SARS-CoV-2 subgenomic transcripts in the viral preparations, as well as several candidate modification sites in the SARS-CoV-2 genomic RNA.

**TABLE 1:** Summary of the SARS-CoV-2 isolates used in this study.

Code used in this manuscript	BEI #	Viral isolate name	Titer (PFU/ml) (Vero E6)	Lineage	GISAID clade	GISAID ID	Clinical presentation/COVID-19 outcome
Viral isolate #1	NR-53517	SARS-CoV-2, Isolate New York-PV09197/2020	1.5 × 10^4^	B.1.3	GH	EPI_ISL_422552	90 years old Male; severe COVID-19 with fatal outcome
Viral isolate #2	NR-53514	SARS-CoV-2, Isolate New York-PV08410/2020	5 × 10^3^	B.1	GH	EPI_ISL_421374	63 years old Male; severe COVID-19 with fatal outcome
Viral isolate #3	NR-52439	SARS-CoV-2, Isolate Chile/Santiago_op4d1/2020	3.25 × 10^4^	A.2	S	EPI_ISL_415661	Patient has respiratory tract infection. History of travel to Europe
Viral isolate #4	NR-53515	SARS-CoV-2, Isolate New York-PV08449/2020	1 × 10^4^	B.1	GH	EPI_ISL_421400	88 years old Female; severe COVID-19 with fatal outcome
Viral isolate #5	NR-52368	SARS-CoV-2, Isolate New York 1-PV08001/2020	2 × 10^4^	B.4	O	EPI_ISL_414476	39 years old Female; history of travel to Iran
Viral isolate #6	NR-53516	SARS-CoV-2, Isolate New York-PV09158/2020	5.75 × 10^4^	B.1.3	GH	EPI_ISL_422525	62 years old Male; severe COVID-19 with fatal outcome

## Results and Discussion

### tRNA-Seq

**FIGURE 1:**
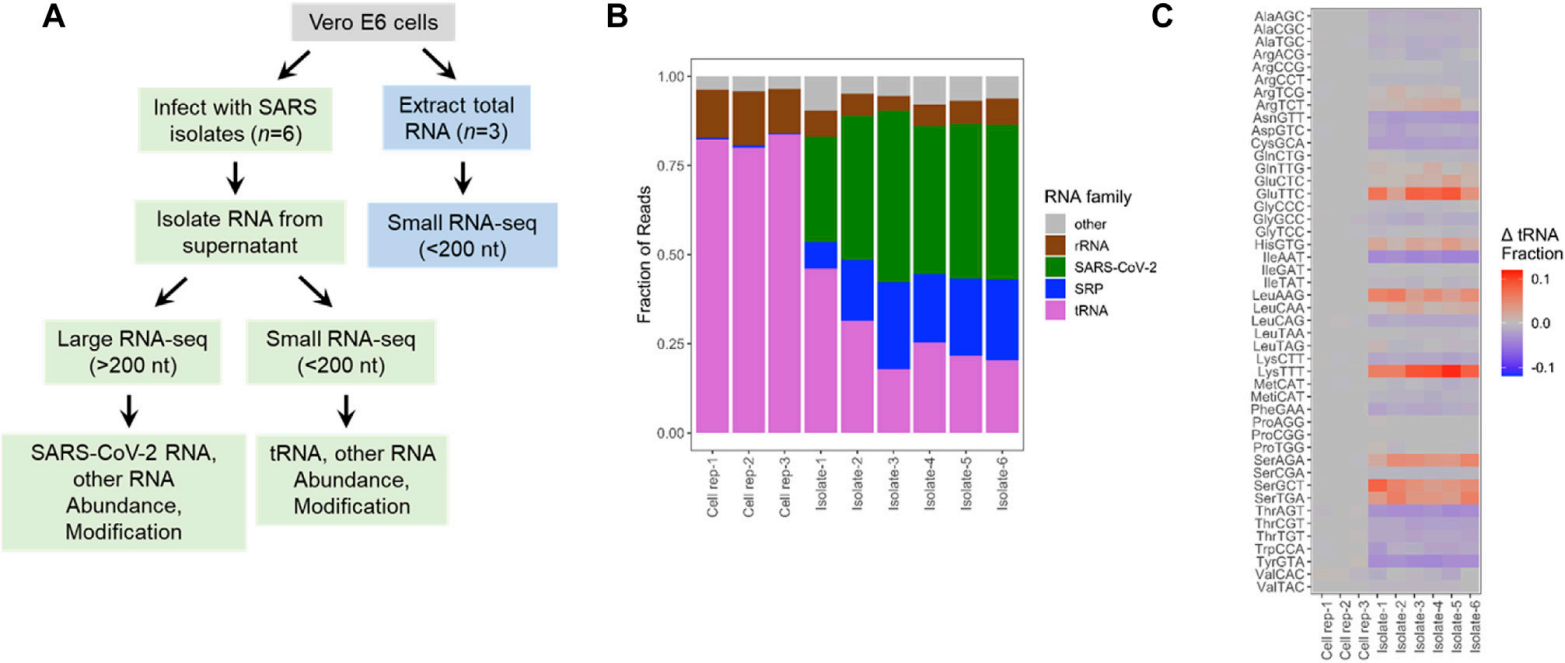
Selective enrichment of small RNAs in SARS-CoV-2 viral preparations. **(A)** Experimental scheme. Vero E6 cells were either infected with SARS-CoV-2 virus isolates from infected individuals (*n* = 6 biological isolates) or uninfected cultures (*n* = 3 biological replicates). Total RNA was extracted from the cells (blue boxes) or only from the cell free viral preparations (green boxes). Small RNA-seq was carried out using total RNA with and without demethylase treatment. Large RNA-seq was carried out with the RNA fraction after the removal of small RNAs of <200 nt, and chemical fragmentation. **(B)** Small RNA-seq results. Vero cell data are mostly tRNA and 5S/5.8S rRNA. Aside from SARS-CoV-2 RNA, virions contain significant portions of tRNA, rRNA, and signal recognition particle (SRP) RNA. **(C)** Enrichment and depletion of specific tRNAs in the cell free viral samples. Shown are the combined reads from all tRNA isodecoders that share the same anticodon. Heatmap shows the abundance of tRNAs for each anticodon subtracted from the mean of control cultures. Subtraction emphasizes the differences among abundant tRNAs. Enriched tRNAs are in red, depleted tRNAs in blue. Top 3 enriched tRNAs are tRNA^Lys^(TTT), tRNA^Glu^(TTC), and tRNA^Ser^(GCT). Top 3 depleted tRNAs are tRNA^Ile^(AAT), tRNA^Tyr^(GTA), and tRNA^Asn^(GTT).

We performed Illumina sequencing starting with total RNA extracted from VeroE6 cells and from SARS-CoV-2 viral preparations cultured on VeroE6 cells (Figure 1A; Supplementary Table S1). To improve efficiency and quantitative assessment of small RNA-seq, defined here as RNA of <200 nucleotides in length, we built two libraries for each sample. The first library was treated with a demethylase mixture (DM) (Zheng et al., 2015) which removed many Watson-Crick face methylations in tRNA that impede reverse transcription in library construction while the second library was left untreated. As described previously, the DM-treated libraries are useful for quantitative assessments of transcript abundance, whereas the untreated samples are useful for modification analysis (Clark et al., 2016). As expected, sequencing reads of the VeroE6 cells mostly mapped to tRNAs, followed by those from 5S to 5.8S rRNA, a small amount of SRP RNA, and others such as spliceosomal RNA (snRNA) and Y RNA (Figure 1B). In the viral preparations, a substantial proportion of reads mapped to viral genomic RNA as expected, and tRNA and SRP RNA are present at almost equally high proportions, followed by a small amount of rRNA (Figure 1B). Although one cannot exclude RNA in exosomes or extracellular RNA not associated with vesicles, our data clearly show RNAs that are differently present in our cell free preparations as compared to those in cells. Our viral preparations used for the sequencing experiments contain high levels of infectious particles outside the cell, strongly suggesting that the sequenced RNA are derived, to a large extent, from cell free virions. For example, our results show a ∼150-fold enrichment of the SRP RNA over tRNA in the viral preparation samples vs. the cell samples, which suggests that we eliminated most if not all of the cellular debris. These results indicate that SARS-CoV-2 virions also package tRNA and SRP RNA in significant proportions.

We next examined the selectivity of packaged tRNA at two levels. Mammalian genomes contain many tRNA isodecoder genes that share the anticodon, but possess different body sequences; all tRNA isodecoders with the same anticodon belong to a single tRNA isoacceptor family (Goodenbour and Pan, 2006; Schimmel, 2018). Abundance of isodecoders was summed for each isoacceptor family and used to calculate the fraction of tRNA reads for each anticodon. The isoacceptor abundance fraction for each sample (three biological replicates of uninfected VeroE6 cells and six distinct viral culture supernatant preparations), was compared to the mean of Vero E6 cells. All three VeroE6 cell replicates were nearly identical, as the heat map shows close to zero values in all cases (Figure 1C). To avoid exaggerated representation of low abundant tRNAs by ratioed comparison, we subtracted the tRNA fraction in each viral preparation to its counterpart in the VeroE6 cells, so that the differences were readily identified for more abundant tRNAs (Figure 1C; Supplementary Table S2). We found several isoacceptor families that are significantly enriched across all six isolates. They include tRNA^Glu^(TTC), tRNA^Lys^(TTT), tRNA^Leu^(AAG), tRNA^Ser^(AGA), tRNA^Ser^(GCT), and tRNA^Ser^(TGA). These results indicate that SARS-CoV2 virions selectively incorporate tRNA isoacceptors.

**FIGURE 2:**
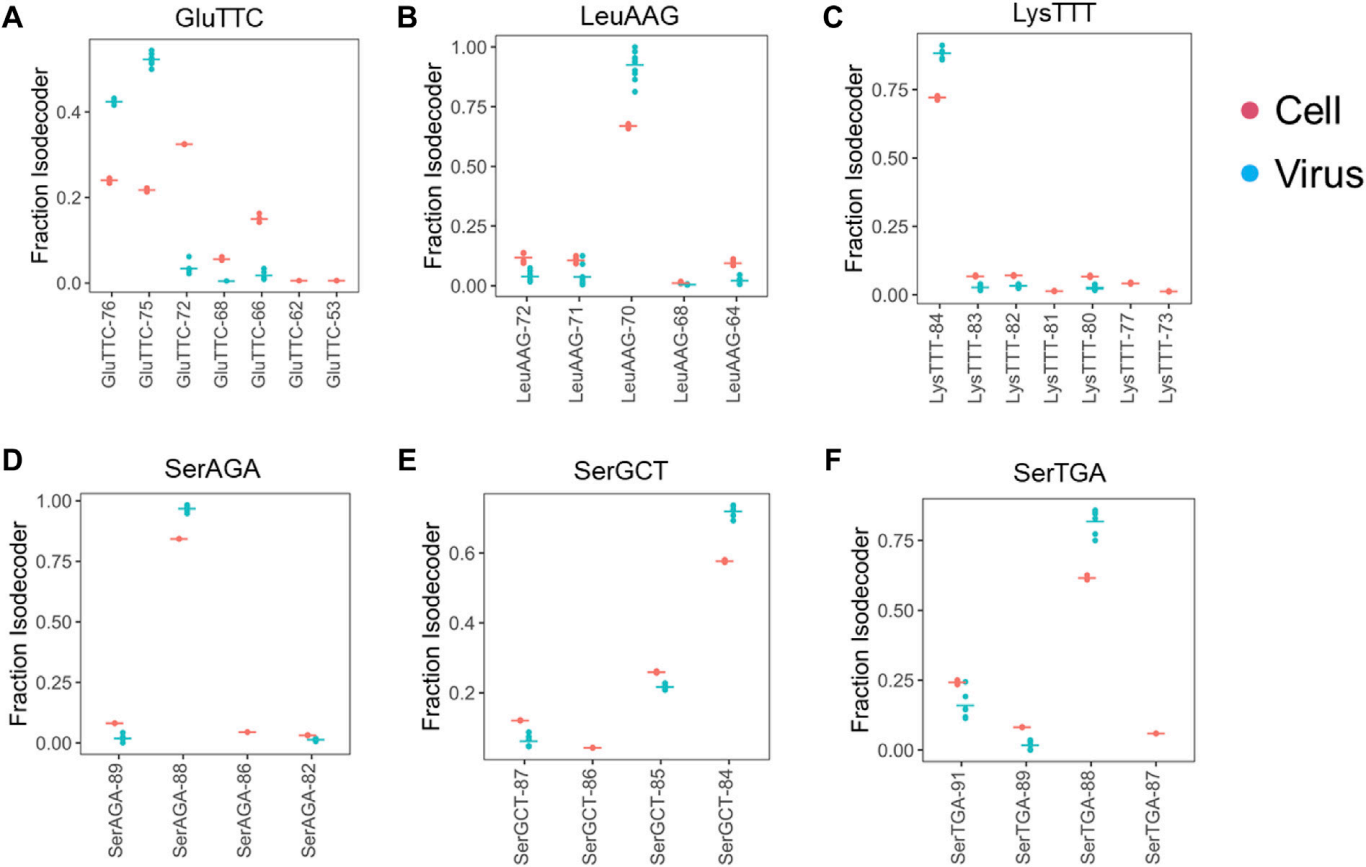
Selective enrichment of tRNA isodecoders in SARS-CoV-2 viral preparations. tRNA isodecoder fractions from uninfected Vero cell (*n* = 3, red) or cell free viral preparations (*n* = 6, blue) are shown. Mean values are shown as a horizontal bar. Isodecoder nomenclature is according to the tRNAScan score of the *Chlorocebus sabaeus* tRNA genes identified in Rfam database. **(A)** tRNA^Glu^(TTC). **(B)** tRNA^Leu^(AAG). **(C)** tRNA^Lys^(TTT). **(D)** tRNA^Ser^(AGA). **(E)** tRNA^Ser^(GCT). **(F)** tRNA^Ser^(TGA).

Our downstream analysis, thus, focused on those six tRNAs enriched in the viral preparations. First, we analyzed the tRNA at the isodecoder level for all six tRNAs. Among the seven tRNA^Glu^(TTC) isodecoders, four could be detected in the viral preparations. However, only two isodecoders represent almost all tRNA^Glu^(TTC) in the viral preparations, even though neither is the most abundant isodecoder in VeroE6 cells (Figure 2A). In contrast, the single dominant tRNA^Leu^(AAG), tRNA^Lys^(TTT), tRNA^Ser^(AGA) isodecoders in cells are also the ones in the viral preparations (Figures 2B–D). For tRNA^Ser^(GCT) and tRNA^Ser^(TGA), two isodecoders each are present at appreciable levels, in each case, the isodecoder at the highest level is also the one in the viral preparations (Figures 2E,F).

**FIGURE 3:**
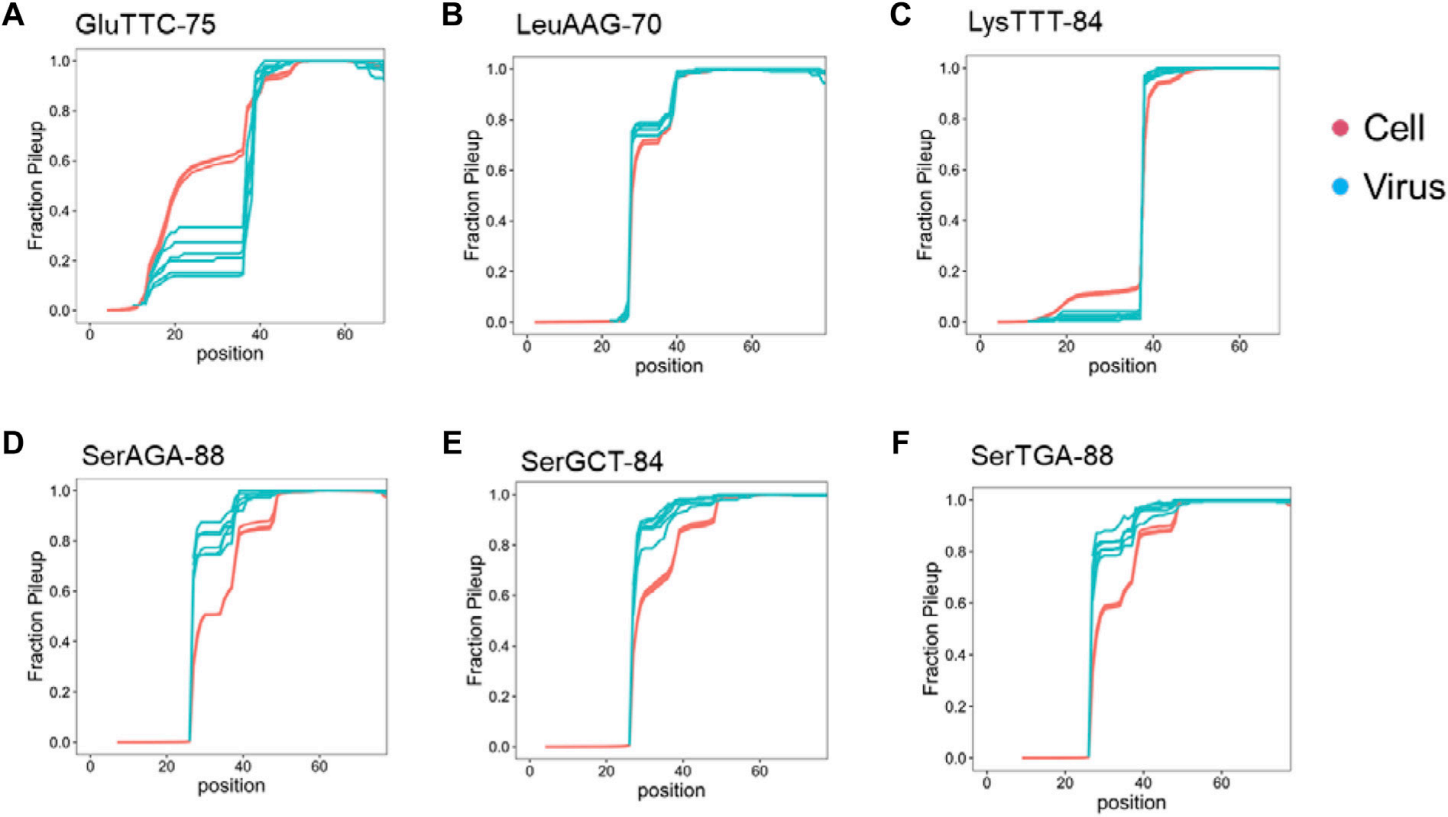
Read pileup of the enriched tRNA isodecoder in SARS-CoV-2 viral preparations. Shown are read pileups of the most abundant tRNA isodecoders in viral isolates (*n* = 6, blue) and their counterparts in uninfected Vero cell (*n* = 3, red). Isodecoder nomenclature is according to the tRNAScan score of the *Chlorocebus sabaeus* tRNA genes identified in Rfam database. **(A)** tRNA^Glu^(TTC). This result is consistent with 3′ tRNA fragment being the dominant form in the viral preparations. **(B)** tRNA^Leu^(AAG). This result is consistent with full-length tRNA in the viral preparations. **(C)** tRNA^Lys^(TTT). This result is consistent with full-length or 3′ tRNA fragment with 5′ end exactly at position 39 in the viral preparations. **(D)** tRNA^Ser^(AGA). The tRNA^Ser^ results are consistent with full-length tRNA in the viral preparations. **(E)** tRNA^Ser^(GCT). **(F)** tRNA^Ser^(TGA).

We examined the read pileup of the most abundant isodecoder in the viral preparations. By experimental design, our tRNA-seq results always start from the 3′ end of the tRNA and show a decline toward the 5′ end with sharp drops at certain tRNA modifications, an expected behavior for full-length tRNAs (Zheng et al., 2015). Three types of results are observed: first, the pileup decreases faster in the viral preparation tRNA compared to the cellular tRNA, this group includes tRNA^Glu^(TTC) (Figure 3A). The pronounced drop of the viral preparation tRNA in the anticodon loop region is consistent with GluTTT-75 in the virion being a 3′ half tRNA fragment with the 5′ end in the anticodon loop, because there is no known RT stopping modifications in this tRNA. In the second type, the read pileup decreases at about the same rate, this group includes tRNA^Leu^(AAG) and tRNA^Lys^(TTT) (Figures 3B,C). The similar drop off is consistent with the tRNA in the viral preparations as the full-length tRNA like those in cells, and the sharp drop offs corresponds to the N2,2-dimethyl-G at position 26 (m^2^_2_G26) in tRNA^Leu^(AAG) which is difficult to remove by the demethylase because it is buried in the tRNA structure (Dai et al., 2017) and 2-methylthio-6-carbamoylthreonine at position 37 (ms^2^t^6^A37) in tRNA^Lys^(TTT) (Machnicka et al., 2013) which does not react with the demethylase. In the third type, the read pileup decreases slower in the viral preparation tRNA, this group includes all three tRNA^Ser^, and the sharp drop offs correspond to the m^2^_2_G26 modification (Figures 3D–F). This result is consistent with the tRNA^Ser^ in the viral preparations having lower modification levels in the anticodon stem-loop region which can include N6-methyl-N6-threonylcarbamoyladenosine at position 37 (m^6^t^6^A37) in tRNA^Ser^ (Machnicka et al., 2013).

**FIGURE 4:**
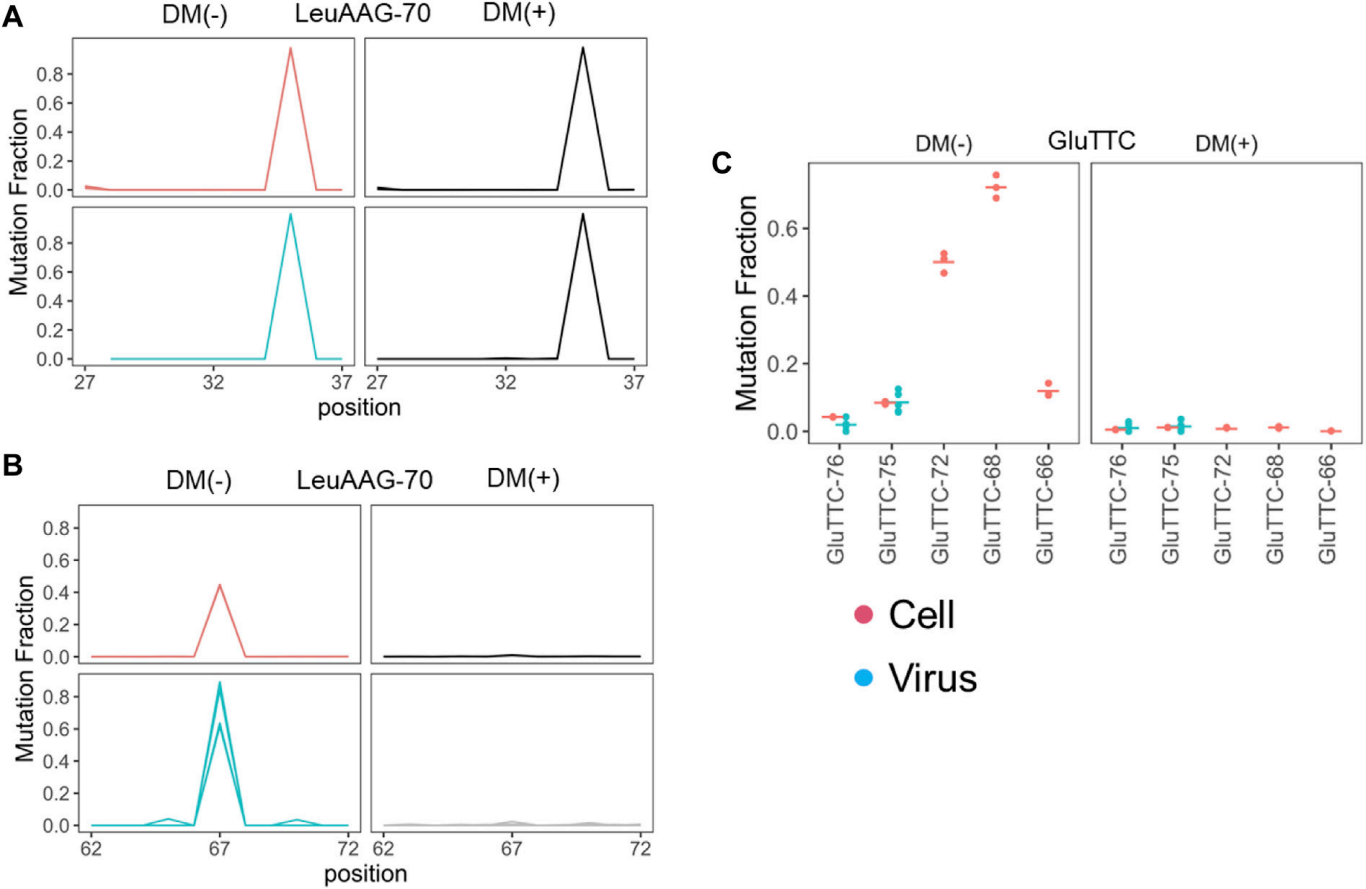
Selective enrichment of tRNA with m^1^A modification profiles. Mutation fractions from uninfected Vero cell (*n* = 3, red) or cell free viral preparations (*n* = 6, blue) are shown. **(A)** Mutation fractions of tRNA^Leu^(AAG) residues around the wobble anticodon position (35 for this tRNA) without (DM-) and with (DM+) demethylase treatment showing the I34 modification. **(B)** Mutation fractions of tRNA^Leu^(AAG) around the residues at position 67 which corresponds to m^1^A58 in the tRNA nomenclature. tRNA^Leu^(AAG) shows higher mutation fraction in the viral preparations, consistent with SARS-CoV-2 selectively packaging m^1^A modified tRNA^Leu^(AAG). **(C)** Mutation fractions of the top five abundant tRNA^Glu^(TTC) isodecoders at position 57 (DM-) which is validated as m^1^A in the T loop upon removal by demethylase treatment (DM+). Isodecoder nomenclature is according to the tRNAScan score of each tRNA^Glu^(TTC) gene. The two isodecoders enriched in the viral preparations are nearly unmodified, corresponding to their counterparts in the Vero cells.

We next looked for RNA modification differences among the tRNAs from cells and viral preparations. In tRNA-seq, certain modifications can be identified by their “mutation” signatures in the sequencing data. Vero cells are derived from *Chlorocebus sabaeus* kidney, their tRNA modifications have not been reported in the literature. We analyzed the mutation signatures of the cellular tRNAs in the libraries without demethylase treatment and validated the methylations using the results from demethylase treatment (Clark et al., 2016) to provide a comprehensive analysis of Vero cell tRNA modifications (Table 2). Overall, the *C. sabaeus* tRNA modification patterns detected by sequencing are very similar to those from the human HEK293T cells (Table 1 in Clark et al. (2016)). A minor difference is the m^2^_2_G26 modification which is present in tRNA^Val^ in *C. sabaeus* Vero cells but not in HEK293T cells. We detected inosine modification at the wobble anticodon position (I34) in all tRNAs that are A34 in the genome which include tRNA^Leu^(AAG) and tRNA^Ser^(AGA) (Figure 4A; Supplementary Figure S1A; Table 2).

**TABLE 2:** Modifications identified in the *Chlorocebus sabaeus* tRNAome.

tRNA^a^	m^1^A58	m^1^G37	I34^b^	m^2^_2_G26	m^1^G9	m^3^C
AlaAGC	X	X^c^	X	X		
AlaCGC	X	X^c^		X		
AlaTGC	X	X^c^		X		
CysGCA	X					
AspGTC	X				X^d^	
GluCTC	X					
GluTTC	X					
PheGAA	X			X		
GlyCCC	X					
GlyGCC	X					
GlyTCC	X					
HisGTG	X	X				
IleAAT	X		X	X		
IleTAT	X			X		
LysCTT	X					
LysTTT	X					
LeuAAG	X	X	X	X		X^e^
LeuCAG	X	X		X		
LeuTAG	X	X		X		
LeuCAA	X	X		X		
LeuTAA	X	X		X		
Met-i	X			X	X	X^f^
Met-e	X					
AsnGTT	X			X	X	
ProAGG	X	X	X		X	
ProCGG	X	X			X	
ProTGG	X	X			X	
GlnCTG	X					
GlnTTG	X					
ArgACG	X		X	X		
ArgCCG	X			X		
ArgTCG	X			X		
ArgCCT	X				X	X^g^
ArgTCT	X				X	X^g^
SecTCA	X					
SerAGA	X		X	X		
SerCGA	X			X		X^h^
SerTGA	X			X		X^h^
SerGCT	X			X		X^h^
ThrAGT	X		X	X	X	X^g^
ThrCGT	X			X	X	X^g^
ThrTGT	X			X	X	X^g^
ValAAC	X		X	X		
ValCAC	X			X		
ValTAC	X			X		
TrpCCA	X	X		X	X	
TyrGTA	X	X		X		

a m^1^A, m^1^G, m^2^_2_G, and m^3^C mutations in sequencing are demethylase sensitive. X = present.

b I34 is not sensitive to demethylase treatment.

c m^1^I37 in tRNA^Ala^.

d m^1^A9 in tRNA^Asp^.

e m^3^C47d (in variable loop of type II tRNA) in tRNA^Leu^(CAG).

f m^3^C20 in tRNA^eMet^.

g m^3^C32 in tRNA^Arg^ and tRNA^Thr^.

h m^3^C32 and m^3^C47d in tRNA^Ser^.

We then compared the mutation levels between the tRNAs enriched in viral preparations and their counterparts in cells. For high confidence analysis we applied a filter of ≥50 read coverage at nucleotide positions of interest. The modification sites passing this filter among the tRNAs in viral preparations only include m^1^A58 (tRNA nomenclature) in tRNA^Leu^(AAG), tRNA^Lys^(TTT), and tRNA^Glu^(TTC), and I34 in tRNA^Leu^(AAG) and tRNA^Ser^(AGA). For tRNA^Leu^(AAG) and tRNA^Lys^(TTT), the mutation fraction at m^1^A58 is higher in the tRNA from the viral preparations than the VeroE6 cell tRNA (Figure 4B; Supplementary Figure S1B), suggesting preferential packaging of m^1^A modified tRNA. Among the tRNA^Glu^(TTC) isodecoders, m^1^A58 level is variable in cells. Only two of the five abundant tRNA^Glu^(TTC) isodecoders have high modification levels in VeroE6 cells, but only isodecoders with low modification fractions are present in the viral preparations (Figure 4C). Since tRNA^Glu^(TTC) in the virions are likely tRNA fragments, this result is consistent with low m^1^A modified tRNA^Glu^(TTC) being the preferred source of tRNA^Glu^(TTC) fragments in cells. tRNAs in cells and in the viral preparations are >90% modified with I34 in both tRNA^Leu^(AGA) and tRNA^Ser^(AGA) (Figure 4A; Supplementary Figure S1A).

### Viral RNA-Seq

**FIGURE 5:**
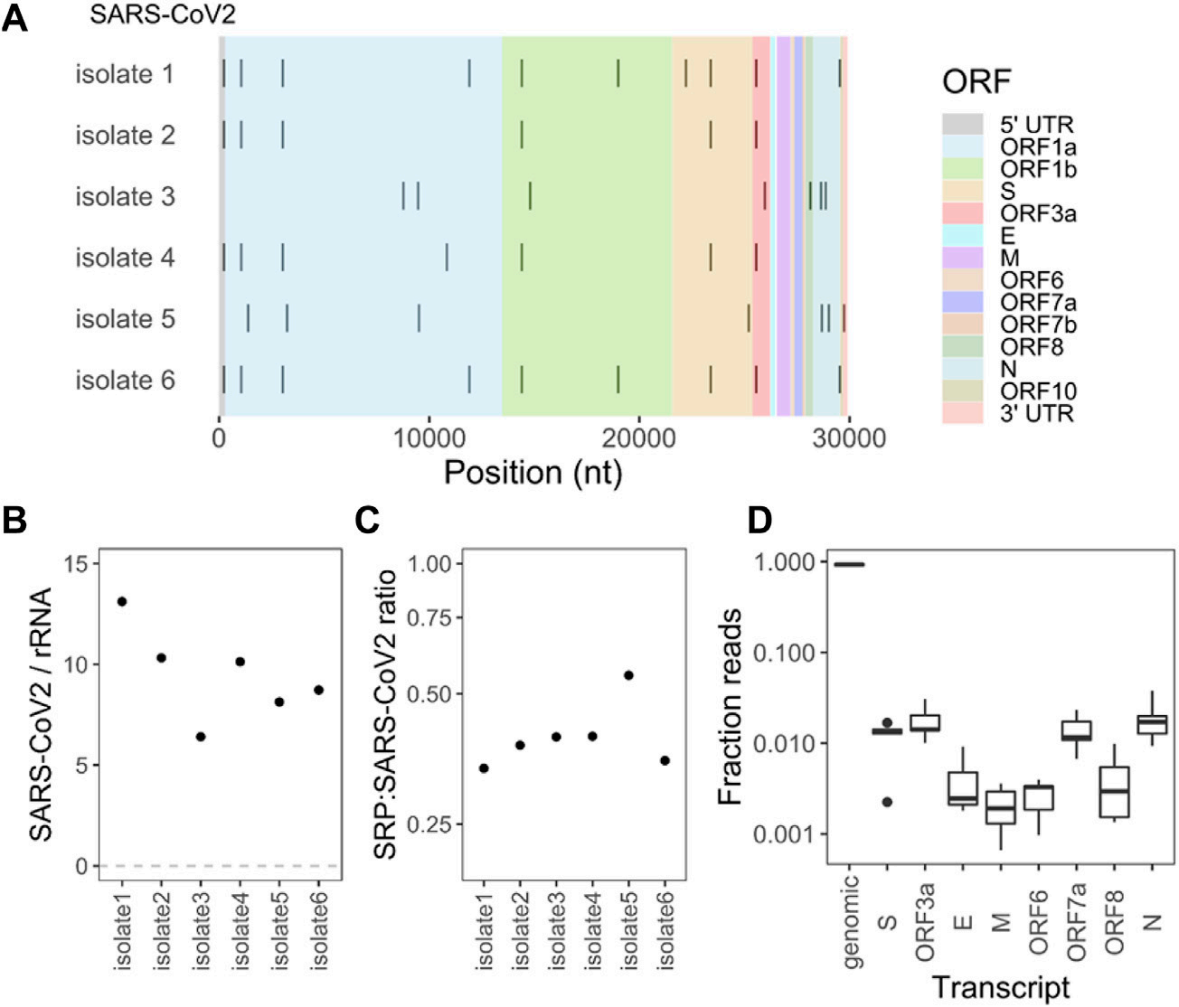
Large RNA sequencing identifies viral sequence variants, subgenomic viral RNAs, and signal recognition particle RNA. **(A)** Single nucleotide polymorphisms (SNPs) for each viral isolate identified by >90% mutation fraction from the Wuhan SARS-CoV-2 reference genome. **(B)** Mapped read count ratio of SARS-CoV2 genomic RNA to large ribosomal RNA (18S and 28S) in the viral preparations. **(C)** Normalized ratio of SRP RNA reads to SARS-CoV-2 genomic RNA reads in the viral preparations using the transcript size of 300 nucleotides for SRP, and 29,903 nucleotides for SARS-CoV-2. **(D)** Relative fraction of reads that bridge the junction between the 5′ leader region and the genomic RNA (set at 1) and between individual subgenomic RNA. Box and Whisker plot for *n* = 6 individual isolates.

We next carried out large RNA-seq (>200 nt) of the viral preparations to characterize the viral RNA and its candidate modifications. We first removed small RNAs by size-selection, followed by chemical fragmentation and library construction. As expected, most of the reads mapped to the SARS-CoV-2 genome (Wuhan reference). We used a mutation threshold of >90% to identify single nucleotide polymorphisms (SNPs) in these samples (Figure 5A). These SNPs did not change upon our enzymatic or chemical treatment described below (data not shown). Our samples are derived from distinct viral isolates obtained from patients infected during the first pandemic wave in the spring of 2020, at the time when most of the circulating SARS-CoV-2 viruses still had only a few sequence changes (Gonzalez-Reiche et al., 2020). We measured the read counts of the SARS-CoV-2 genomic RNA and the 18S + 28S rRNA in our viral preparations (Figure 5B). We found an average ratio of SARS-CoV-2/rRNA of ∼9.5, or a molar ratio of SARS-CoV-2/rRNA of ∼2. Given that the cellular ribosomes and viral particles produced upon infection (Sender et al., 2021) is higher than 100:1, our results show a >200-fold enrichment of SARS-CoV-2 viral RNA over rRNA in our culture supernatant preparations.

We also measured the ratio of reads from the SRP RNA, which was the most abundant RNA in the viral preparations in the small RNA-seq data. We counted the read coverage for the six SARS-CoV-2 genomes and those mapping to the *C. sabaeus* SRP from the Rfam database (Kalvari et al., 2021). We then used a normalization factor that accounts for the length difference of SARS-CoV-2 genome (29,903 nt) and SRP RNA (300 nt) to get a ratio of SRP to SARS-CoV-2 RNA (Figure 5C). We obtained a ratio of .3–.55 for the six viral preparations. Given the intermediate size of the SRP RNA that may incur losses during the size-selection step, we estimate that a virion contains up to one SRP RNA transcript.

We also asked whether our cell free viral preparations contain SARS-CoV-2 subgenomic RNA. Subgenomic RNAs are generated in the infected cells by joining a 5′ leader sequence to each of the SARS-CoV-2 protein coding genes (Supplementary Figure S2). We found 2,000–3,000 reads that cover the junction region of the 5′ leader with Orf1a, which corresponds to the full-length SARS-CoV-2 RNA. Normalizing the reads covering other genes to this junction, we found up to 2% of subgenomic RNAs containing S, Orf3a, Orf7a, and E genes in all six isolates (Figure 5D). Altogether, up to 10% of the viral RNAs in the cell free viral preparations are subgenomic RNAs. This low level is consistent with our viral preparations comprising primarily of SARS-CoV-2 virions, and most virions in the culture containing the full-length genomic RNA. The subgenomic RNAs may represent in part virions devoid of the full-length genomic RNA (i.e., defective viral particles).

**FIGURE 6:**
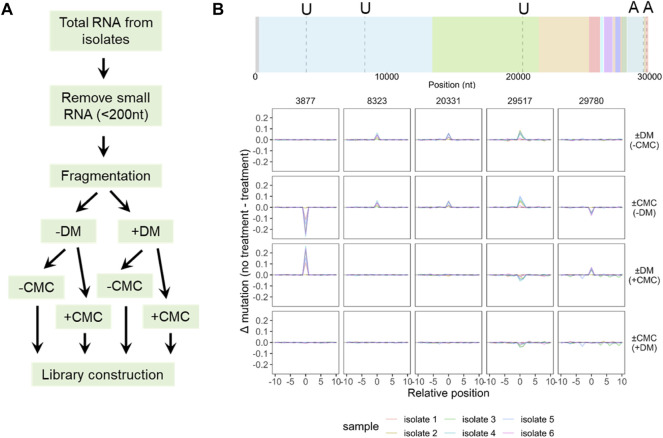
Large RNA sequencing identifies candidate SARS-CoV-2 modifications. **(A)** Scheme of modification detection. An enzyme treatment (DM) and a chemical treatment (CMC) are added before library construction, producing four combinations for each sample. **(B)** Candidate modifications from comparing four treatment conditions. Site locations are indicated in a dashed line, nucleotide identity indicated on top of the SARS-CoV-2 gene map. Data from top to bottom: with and without demethylase only (±DM, -CMC); with and without CMC only (±CMC, -DM), with and without demethylase, also CMC (±DM, +CMC); with and without CMC, also DM (±CMC, +DM). Positions with mutations >5% in at least 4 of the 6 isolates but excluding the SNP positions in Figure 5A are shown.

Finally, we carried out experiments to identify novel modifications of SARS-CoV-2 vRNA. To facilitate this identification, we added two consecutive steps in the large RNA library construction, one with demethylase treatment (DM), and the other with 1-cyclohexyl-(2-morpholinoethyl)carbodiimide (CMC) treatment (Figure 6A). Demethylase treatment generally detects Watson-Crick methylations such as m^1^A in our tRNA studies, whereas CMC treatment is a standard procedure for detecting pseudouridine (Ψ) modification in mRNA (Gilbert et al., 2016). We first carried out a threshold analysis in four pairwise comparisons of ±DM, ±CMC, ±DM (plus CMC treatment), ±CMC (plus DM treatment) using the filters for mutation difference of 5%, stop difference of 15%, deletion difference of 2%, and insertion difference of 2%. We filtered next the candidate sites from the threshold analysis to only include those that show the same signature change in at least four of the six viral preparations. Only the mutation signature of five sites passed these two filters (Figure 6B). These sites fall into four groups: 1) U8323 and U20331 are DM-sensitive without CMC, CMC-sensitive without DM, not detected in DM with CMC, nor in CMC with DM. These two sites may represent N3-methyl-U (m^3^U) derivatives. 2) A29517 is DM-sensitive without CMC, CMC-sensitive without DM, the mutation signals are reversed in DM with CMC, or in CMC with DM. This site may represent N1-methyl-A (m^1^A) derivatives. 3) U3877 is DM-insensitive without CMC, CMC-sensitive without DM, DM-sensitive with CMC, not detected in CMC with DM. This site may represent N1-methyl or N3-methyl-pseudouridine (m^1^Ψ, m^3^Ψ) derivatives. 4) A29780 is DM-insensitive without CMC, CMC-sensitive without DM, DM-sensitive with CMC, not detected in CMC with DM. We do not recognize an A modification at this time that would generate such signatures. No stop signature was observed for any of these five sites (Supplementary Figure S3).

It is intriguing to note that these five candidate modification sites in SARS-CoV-2 do not follow the expected pattern of changes for well-characterized RNA modifications. In all five cases, the mutation levels are at most 20%, which may reflect either low levels of modification at these sites or under-counting the modification levels because of the unknown nature of these modifications. Future studies are needed to pinpoint the exact nature of these modifications.

## Concluding Remarks

In this work, we identified selective enrichment of host tRNAs and SRP RNA in cell free SARS-CoV-2 viral preparations, differences in tRNA modification between the tRNA in viral preparations and in cells, and candidate modification sites in the packaged SARS-CoV-2 genomic RNA. We estimate that a SARS-CoV-2 virion contains up to one molecule of SRP RNA. Given the roughly equivalent read counts of SRP and tRNA in the viral preparation (Figure 1B), and full-length tRNAs are approximately one fourth in size to SRP RNA, we estimate that a SARS-CoV-2 virion also contains up to four tRNA molecules.

How and why specific tRNAs and the SRP RNA are enriched in virions remains unclear. Packaging of the HIV primer tRNA^Lys^(TTT) is facilitated by the host lysyl-tRNA synthetase (LysRS) and gag protein interaction (Cen et al., 2002). As a consequence, tRNA^Lys^(CTT) is also packaged at similar levels. In our case, however, tRNA^Lys^(CTT) was not enriched in the virions. On the other hand, the enrichment of multiple tRNA^Ser^ isoacceptors may go through this mechanism of facilitating tRNA packaging through host seryl-tRNA synthetase (SerRS) and SARS-CoV-2 protein interactions. Retroviruses also package a large amount of SRP RNA into virions (Eckwahl et al., 2015; Eckwahl et al., 2016). Our results add SARS-CoV-2 to this list.

Cells release tRNA fragments into extracellular medium in many ways (Torres and Martí, 2021). tRNA modifications influence tRNA fragment biogenesis, and the secreted tRNA fragments often match the abundance profiles of those fragments in cells. For the tRNA^Glu^(TTC) fragment, its levels and m^1^A58 modification profile match in the viral preparations and in cells. However, the full-length tRNA^Leu^(AGA) and tRNA^Lys^(TTT) in the viral preparations have higher m^1^A58 levels than their counterparts in cells. M^1^A58-modified tRNA can interact differently with cellular components such as eEF1A compared to the hypo-modified tRNA (Liu et al., 2016). The higher tRNA m^1^A58 level in the viral preparations may be related to their enhanced interaction with viral proteins. We also identified candidate modifications in the SARS-CoV-2 genomic RNA. It is surprising that none of the five modification sites described here could be readily assigned to well characterized modifications, just like those sites reported by nanopore sequencing (Kim et al., 2020). Viral RNA modification studies have only taken off recently and future studies will be needed to reveal the chemical nature and the functional consequences of these modifications.

This proof-of-concept study was performed using the viral isolates cultured early in the pandemic (March/April 2020). In future work, we will produce larger amounts of SARS-CoV-2 isolates representative of the early circulating strains as well as the viral variants of concern that have dominated most of the pandemic in 2021. We will culture these isolates on ACE2-expressing human cells rather than on African green monkey VeroE6 cells. These studies will firmly establish the dependency of host RNA packaging on SARS-CoV-2 variants and on host cell source.

## Methods

### SARS-CoV-2 Isolates

Residual nasopharyngeal swab specimens were collected after completion of the diagnostic process as part of the Mount Sinai Pathogen Surveillance Program. To culture SARS-CoV-2 isolates, .1 ml of viral transport media was inoculated into one well of a six-well plate seeded with a confluent monolayer of VeroE6 cells. Culture supernatants were harvested when CPE (cytopathic effect) became visible, aliquotted, and stored at −80°C. All work related to SARS-CoV-2 culture was performed in a BSL3 biocontainment facility by trained personnel and in accordance with the research registration approved by the Institutional Biosafety Committee (IBC).

We cultured six distinct SARS-CoV-2 isolates representing the early lineages of the pandemic (Table 1). After isolation of the clinical isolates on VeroE6 cells, we determined the infectious viral titers for each of the viral culture supernatants by plaque assay (Table 1). All six isolates displayed medium plaque phenotypes. We shared aliquots of the viral stocks analyzed in this study with the NIH BEI repository in the early summer of 2020 (see Table 1 for specifics).

### RNA Isolation

The viral culture supernatants were spun at 3,000 rpm for 10 min to remove particulates. vRNA from each viral preparation and total RNA from VeroE6 cells was performed using QIAamp Viral RNA Kits (Qiagen) following the manufacturer’s instructions. We did not filter the supernatants as that could result in some viruses being absorbed to the membrane and/or causing a loss of viral infectivity.

### RNA Library Construction and Sequencing

#### Small RNA-Seq

The procedure was adapted from DM-tRNA-seq (Zheng et al., 2015) with the following modifications for tRNA deacylation: 10 µl of total RNA from viral culture supernatants or uninfected cells (containing up to a maximum of 1 µg of total RNA, as measured by NanoDrop) were deacylated by adding 5 µl of 100 mM Na_2_B_4_O_7_, pH 9.5 (final concentration: 33.3 mM) and incubated at 37°C for 30 min. To the deacylated samples, 5 µl of a 3′-end clean-up mixture (200 mM Tris-HCl, pH 6.8, 40 mM MgCl_2_, and 4 U/µl T4 PNK [NEB]) were added and incubated at 37°C for 20 min, and then heat inactivated at 65°C for 10 min. We used superscript IV RT in this work.

#### Large RNA-Seq

1 µg of total RNA from infected Vero cell culture preparations was diluted to 50 µl in microcentrifuge tubes. Zymo RNA Clean and Concentrator-5 columns (R1016, Zymo) were used to remove small RNAs ≤200 nt by following the manufacturer’s protocol. The large RNA (>200 nt) was eluted in 18 µl RNase free water. PCR machine was preheated at 95°C for RNA fragmentation experiment. Eluted RNA was then transferred to PCR tubes, 2 µl RNA fragmentation buffer (E6150S, NEB) was added to each tube and mixed well. Samples were incubated at 95°C on PCR machine for 6 min (target fragmentation is 200–500 nt) followed by putting on ice immediately. 2 µl RNA fragmentation stop buffer (E6150S, NEB) was added to each tube and mixed well. Samples were transferred to microcentrifuge tubes and diluted to 50 µl. Zymo RNA Clean and Concentrator-5 columns were used to cleanup RNA (>200 nt). RNA was eluted in 8 µl RNase free water. 1 μl T4 PNK buffer and 1 µl 10 U/μl T4 PNK were added to each tube and mixed well. The samples were incubated at 37°C for 30 min to repair RNA fragment 3′ ends. The samples were spun down and incubated at 75°C for 10 min followed by immediately putting on ice to inactivate T4 PNK. All samples (∼10 µl reaction mixture each) were used to build the libraries using bead-based library construction methods developed in our lab. Briefly, the first adaptor ligated RNA fragments were captured on the beads and pooled. The beads were then split into two equal parts for ± demethylase treatment. After demethylase treatment, the beads were split again to two parts for −CMC and +CMC treatment (1:1.5 ratio) (Zhang et al., 2019). The ± demethylase and ±CMC treated beads were then used to continue the library construction.

### Sequencing Data Analysis

Reference RNA sequences from *Chlorocebus sabaeus* that included non-coding RNA and tRNA were downloaded from Rfam database (https://rfam.xfam.org/, Kalvari et al., 2021). *C. sabeaus* tRNA sequences from Rfam were processed through tRNAScan-SE (http://lowelab.ucsc.edu/tRNAscan-SE/, Lowe and Chan, 2016), only sequences with high confidence (i.e., tRNAScan score ≥50) were used as reference. Following this, tRNA sequences were appended by adding CCA at their 3′ end as well as removing intron sequences (Supplementary Table S1). These processed *C. sabeaus* cytosolic tRNA, mitochondrial tRNA, and non-coding RNA sequences such as SRP from Rfam were combined with the Wuhan SARS-CoV-2 genome sequence (MN908947.3) to generate a custom reference database.

Raw reads following sequencing were designated reads 1 and reads 2 and were merged together using bbmerge.sh present within the bbmap package (https://github.com/BioInfoTools/BBMap), which results in merged fastq files. These merged fastq were converted to fasta file format using reformat.sh present within the bbmap package. These fasta files were aligned to our custom reference genome using bowtie2 (http://bowtie-bio.sourceforge.net/bowtie2/index.shtml, Langmead et al., 2009) with the following parameters: -f -p 10 --local —no-unal. The aligned reads were then used to determine RNA sequence abundance using custom python script. RNA modifications were detected based on aligned reads using samtools sort (http://www.htslib.org/, Li et al., 2009) feature sort the reads in a bam file format. Then IGVtools count (https://software.broadinstitute.org/software/igv/igvtools) count feature was utilized to output a wig files using the following parameters: -z 5 -w 1 -e 250 —bases. The resulting wig files were processed using a custom python script to identify nucleotide mutations as well as coverage of aligned reads.

## Data Availability

The datasets presented in this study can be found in online repositories. The names of the repository/repositories and accession number(s) can be found below: https://www.ncbi.nlm.nih.gov/, GSE182883.
