# Supplementary material for: Profiling Selective Packaging of Host RNA and Viral RNA Modification in SARS-CoV-2 Viral Preparations
Source: Front Cell Dev Biol. 2022 Feb 3;10:768356. doi: 10.3389/fcell.2022.768356 (PMC8851031; doi:10.3389/fcell.2022.768356)
Supplement: Supplementary file 2 [file Presentation1.pdf]

Pena et al. **Profiling selective packaging of host RNA and viral RNA modification inSARS-CoV-2 viral preparations**

Supplemental Figures

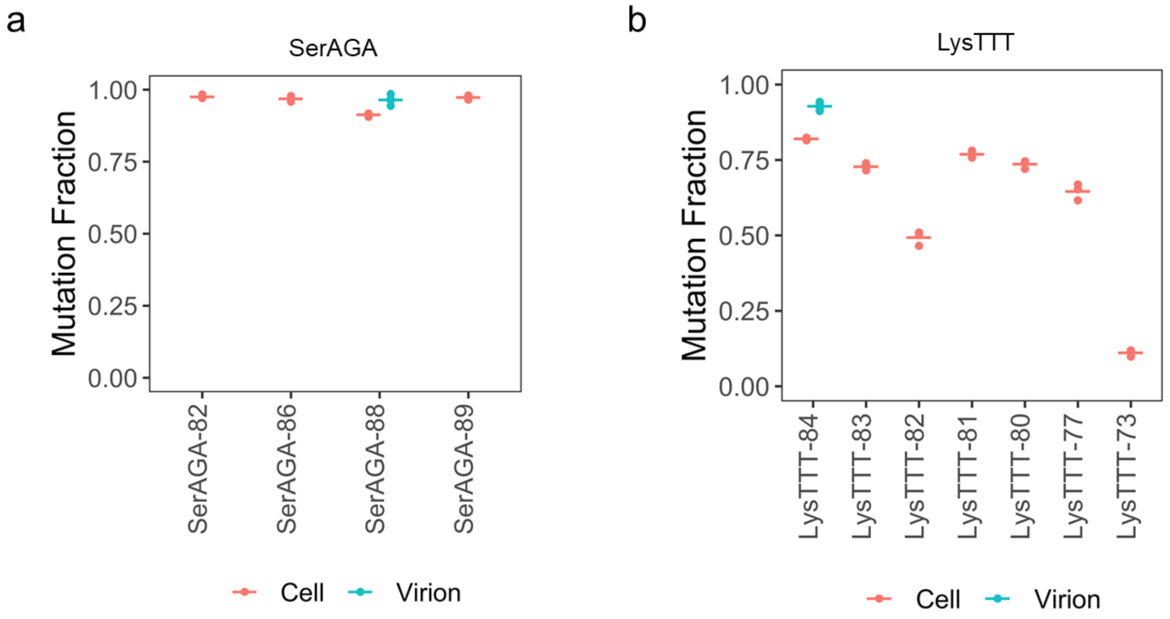

Figure S1: Selective enrichment of other tRNA with modification profiles.
(a) Mutation fractions of $tRNA^{Ser}$(AGA) isodecoders from uninfected Vero cell (*n*=3, red) or viral preparations (*n*=6, blue) of the wobble anticodon position (I34).

(b) Mutation fractions of $tRNA^{Lys}$(TTT) isodecoders of position m^1A58.

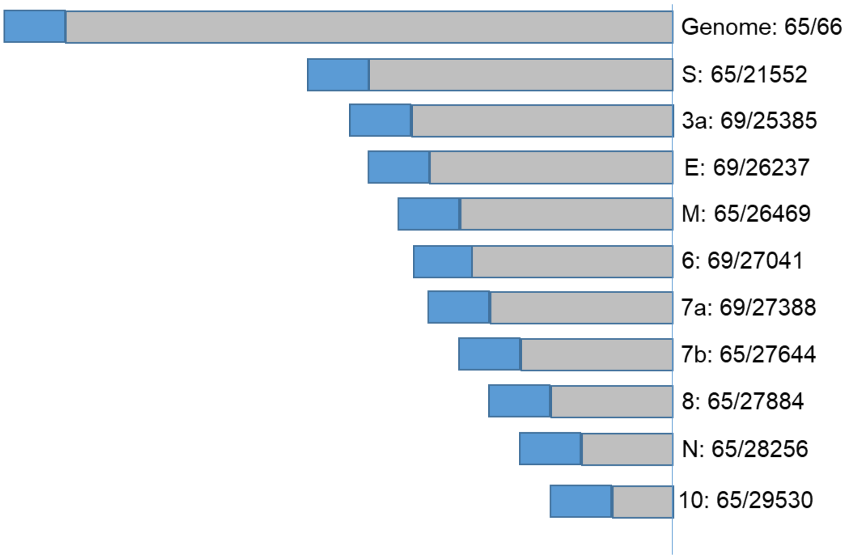

Figure S2: Subgenomic junction sites used in our large RNA analysis. Residue numbers are according to the Wuhan SARS-CoV-2 reference genome. Residues linking the 5’ leader and the subgenomic regions are indicated.

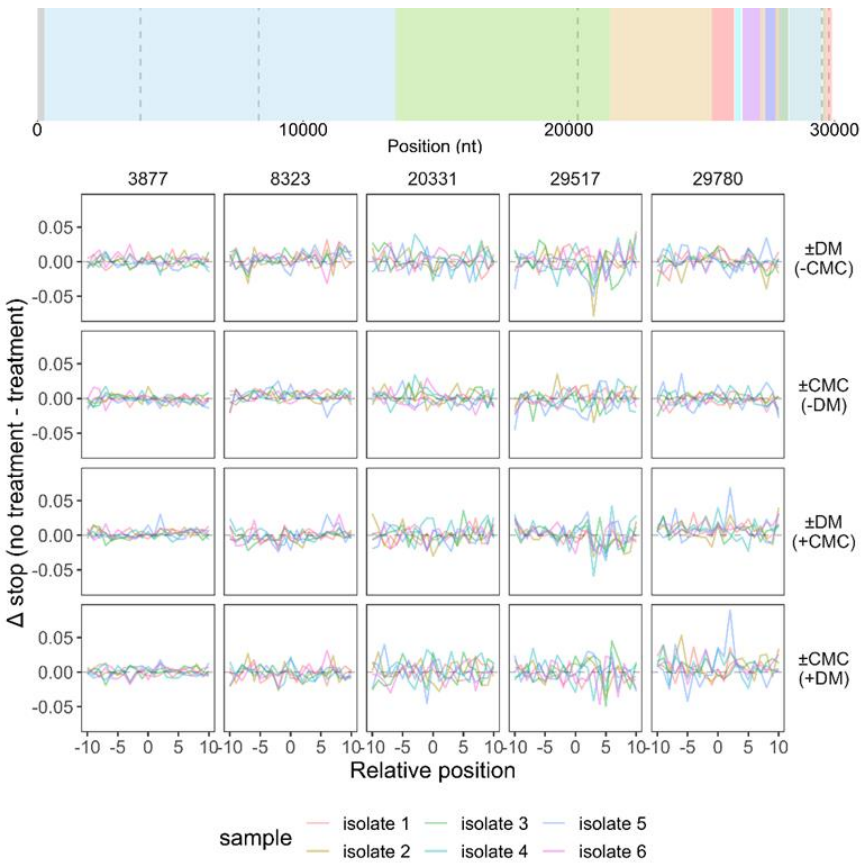

Figure S3: Stop signatures of the five sites in Fig. 6b.
